# The future of driving pressure: a primary goal for mechanical ventilation?

**DOI:** 10.1186/s40560-018-0334-4

**Published:** 2018-10-04

**Authors:** Hiroko Aoyama, Yoshitsugu Yamada, Eddy Fan

**Affiliations:** 1 Department of Anesthesiology, Graduate School of Medicine, The University of Tokyo, 7-3-1 Hongo, Bunkyo-ku, Tokyo, 113-0033 Japan; 2 Interdepartmental Division of Critical Care Medicine, University of Toronto, 585 University Avenue, Toronto, Ontario M5G 2N2 Canada

**Keywords:** Driving pressure, Lung protective mechanical ventilation, Acute respiratory distress syndrome, Mortality, Intensive care units, Clinical practice guideline

## Abstract

**Background:**

Management of patients with acute respiratory distress syndrome (ARDS) remains supportive with lung protective mechanical ventilation. In this article, we discuss the physiological concept of driving pressure, current data, ongoing trials, and future directions needed to clarify the role of driving pressure in patients with ARDS.

**Body:**

Driving pressure is the plateau airway pressure minus PEEP. It can also be expressed as the ratio of tidal volume to respiratory system compliance, indicating the decreased functional size of the lung observed in patients with ARDS (i.e., baby lung). Driving pressure as a strong predictor of mortality in patients with ARDS is supported by a post hoc analysis of previous randomized controlled trials and a subsequent meta-analysis. Importantly, the meta-analysis suggested targeting driving pressure below 13–15 cmH2O. Ongoing clinical trials of driving pressure in patients with ARDS focus mainly on physiological rather than clinical outcome but will provide important insights for the design of future clinical trials.

**Conclusion:**

Currently, no definite clinical recommendations on the routine use of driving pressure in patients with ARDS can be made, as the available data are hypothesis-generating. Randomized controlled trials are needed to evaluate the efficacy of a driving pressure-based ventilation strategy.

## Commentary

The main aim of this article is to review the concept of driving pressure in adult and mechanically ventilated patients with acute respiratory distress syndrome (ARDS).

## Background

A recent global epidemiologic study reported that 10.4% of total ICU admissions and 23.4% of all intubated patients were diagnosed as having ARDS, and mortality remains high [1]. Over the last 50 years [2], research has led to advances in mechanical ventilation that has improved survival among patients with ARDS [3]. However, it is also acknowledged that mechanical ventilation can also lead to ventilator-induced lung injury (VILI) [4]. Therefore, the current evidence-based management goal is to deliver lung protective ventilation with lower tidal volumes (between 4 and 8 mL/kg predicted body weight [PBW]) and limited airway pressures (below 28–30 cmH_2_O) in order to mitigate VILI in patients with ARDS [5, 6].

## Driving pressure (see Table 1 for related terminology)

In patients without spontaneous breathing efforts (i.e., sedated and/or paralyzed on controlled mechanical ventilation), the driving pressure of the respiratory system is defined as the difference between plateau pressure and positive end-expiratory pressure (P_plat_-PEEP), and can also be expressed as the ratio of tidal volume to respiratory system compliance (Vt/C_rs_) [7]. The potential importance of driving pressure in patients with ARDS was first recognized in 1998 [8]. More recently, a secondary analysis of previous randomized controlled trials (RCTs) of mechanical ventilation in ARDS patients demonstrated that driving pressure is the variable that is most strongly associated with mortality [7]. This finding was later confirmed in a systematic review and meta-analysis of driving pressure in patients with ARDS [9]. Moreover, the results of the meta-analysis suggested targeting driving pressure below 13–15 cmH_2_O, although it remains unclear how to best reduce the driving pressure as part of a ventilatory strategy at the bedside [9].

**Table 1:** Box glossary of driving pressure and lung mechanics in ARDS

▪ Driving pressure (DP): the change in airway pressure during a tidal breath. ◦ DP = P_plat_ − PEEP ◦ C_rs_ = V_t_/(P_plat_ − PEEP) = V_t_/DP ◦ DP = V_t_/C_rs_ Thus, DP represents the tidal volume corrected for the patient’s respiratory system compliance. *Transpulmonary driving pressure (DP_L_): DP_L_ represents dynamic stress that takes into account the chest wall elastance whereas DP represents total (dynamic plus static) stress. ◦ DP_L_ = (P_plat_ − PEEP) − (plateau esophageal pressure − end-expiratory esophageal pressure) ▪ Static compliance of the respiratory system (C_rs_): the compliance measured during periods without gas flow. C_rs_ is strongly correlated with the volume of the baby lung [13, 14] ▪ Cyclic lung strain: the magnitude of lung deformation (change of volume) during each ventilator cycle [11] ▪ Lung stress: the applied force to lungs during ventilation = transpulmonary pressure [11] ▪ “Baby lung”: the concept that represents the reduced functional lung volume typically observed among patients with ARDS [13]	

Conversely, in the presence of spontaneous breathing efforts, the negative change in pleural pressure generated by spontaneous breathing becomes additive to the distending pressure; therefore, driving pressure may be underestimated without considering these efforts. In these circumstances, the plateau pressure should be measured using a brief inspiratory hold (i.e., when no flow on volume control mode) to calculate actual driving pressure [10]. Although a recent study demonstrated that the spontaneous breathing can be injurious for both the lungs and diaphragm [11], the effects of driving pressure on clinical outcomes in the context of spontaneous breathing remain uncertain.

Since driving pressure is a way of representing the tidal volume adjusted for the respiratory system compliance, one reason that lower driving pressure may be associated with lower mortality may be due to a resultant reduction in cyclic lung stretch/inflation during mechanical ventilation [12]. This hypothesis is supported by the strong correlation between cyclic stretch, VILI, driving pressure, and survival in patients with ARDS—with driving pressure having a stronger association than the unadjusted tidal volume [7]. Furthermore, Chiumello et al. reported that driving pressure was significantly associated with lung stress (transpulmonary pressure) [13]. Thus, driving pressure represents the stress applied to the lungs, and adjusting tidal volume according to driving pressure rather than to predicted body weight may lead to better outcomes for patients, especially those with severely injured lungs. Driving pressure may also be used as a tool to help set PEEP, where lung recruitment and reduced inhomogeneity of stress (applied force to lungs) and strain (change of lung volume) with higher levels of PEEP lead to improved lung compliance and reduced driving pressure. Finally, in the setting of marked lung inhomogeneity (e.g., severe ARDS) which may further exacerbate the risk of VILI (due to stress raisers), the potential benefit of lower driving pressure may be even greater [14].

## Future research on driving pressure

The concept of driving pressure seems intuitive and clinically reasonable given the physiological link to the small volume of aerated lung available for ventilation (“baby lung”) [15, 16]. However, the studies to date are hypothesis-generating and there is currently insufficient data to support the routine clinical use of driving pressure. First, prospective studies are needed to compare the predictive validity of driving pressure as compared to other ventilatory valuables. In addition, transpulmonary driving pressure (the difference between end-inspiratory transpulmonary pressure and end-expiratory transpulmonary pressure) better represents the pressure that actually applied to the lungs and may more accurately reflect the potential influence for VILI especially among patients with severe ARDS. Thus, the use of esophageal manometry to compute transpulmonary pressure is worthy of consideration in future studies. Such studies need to ascertain the optimal target of driving pressure and the subgroup of ARDS patients who could benefit most from ventilatory strategies targeting driving pressure [9]. Second, pilot studies demonstrating the safety and feasibility of a driving pressure-targeted strategy are needed before larger, confirmatory RCTs are conducted. Specifically, we need a better understanding of how a protocol targeting driving pressure can be implemented by bedside clinicians through the adjustment of tidal volume or other ventilatory variables. Finally, well-designed RCTs will be required to evaluate the potential efficacy of a driving pressure-based ventilation strategy as compared to the current standard of care (i.e., lung protective ventilation) [5, 6, 9]. We believe that RCTs evaluating therapeutic interventions in patients with ARDS should have mortality as the primary outcome, but long-term outcomes (e.g., functional status, quality of life) should also be evaluated prospectively. To start, these studies should focus on patients with severe ARDS who typically would not have spontaneous breathing efforts due to deep sedation and paralysis, but subsequent investigations of the impact of driving pressure in the presence of spontaneous breathing efforts will be required. Specifically, initial studies of driving pressure during spontaneous breathing may focus on the physiological and biochemical effects on the lungs and diaphragm.

## Ongoing trials

Currently, there are few clinical trials of driving pressure in ARDS patients listed in clinical trial registries and recruiting patients (Table 2). *ClinicalTrials.gov*, European Union registry, Japanese registries network, ISRCTN, and Australian New Zealand Clinical Trials Registry were searched with term “driving pressure” on July 1, 2018. The DRiving pressure for Optimization of Positive end-expiratory pressure [DROP – Trial ID: ACTRN12618000554268] is an uncontrolled clinical trial in France to determine the best PEEP based on oxygenation (i.e., PaO_2_/FIO_2_ ratio) or driving pressure in patients with ARDS. Patients receive a decremental PEEP trial as part of standard care—maximum PEEP is initially achieved for a plateau pressure < 30 cmH2O, then PEEP is set to 15, 10, and 5 cmH_2_O. Arterial blood gases and driving pressure are measured at each PEEP level. PEEP according to best oxygenation (PaO_2_/FIO_2_ ratio) and the lowest driving pressure will be recorded and compared. Another study is the Does Automated Closed-Loop Ventilation Reduce the Driving Pressure Levels in Patients With ARDS [AiRDRoP—Trial ID: NCT03211494], a randomized crossover trial comparing driving pressure during automated closed-loop ventilation with conventional lung protective ventilation in patients with moderate or severe ARDS. Automated closed-loop ventilation is an extension of closed-loop ventilation, where ventilation and oxygenation are automatically adjusted according to the patient’s work of breathing, end-tidal CO2, and the ARDS Network PEEP-FIO_2_ table [17]. Finally, the Driving Pressure Limited Ventilation for Patients With ARDS [ART-2—Trial ID: NCT02365038] is a multicenter pilot RCT investigating the feasibility of a driving pressure-limited ventilatory strategy in comparison with the ARDS Network strategy in patients with ARDS. In the intervention arm, the tidal volume will be adjusted between 3 and 8 mL/kg PBW to achieve a target driving pressure of 13 cmH_2_O, without a limit on the plateau pressure. The primary outcome is the mean driving pressure between day 1 and day 3 after randomization. This trial will provide more information about how driving pressure-targeted strategy can be carried out at the bedside through the adjustment of tidal volume. Although these trials are focused on physiological outcomes, the results will provide important insights on how to design and conduct future trials of driving pressure in patients with ARDS, evaluating its effect on patient-important outcomes.

**Table 2:** Summary for ongoing trials of driving pressure in ARDS patients

Trial name (trial number)	Trial design	Primary Investigator (country)	Intervention arm	Control arm	Primary outcome	Phase (June 2018)
DRiving pressure for Optimization of Positive end-expiratory pressure (DROP) [ACTRN12618000554268]	An uncontrolled clinical trial	Stephan Francois (France)	Decremental PEEP as part of standard care, where maximum PEEP for a plateau pressure < 30 cmH2O, then PEEP is set to 15, 10, and 5 cmH2O	Not applicable	Best PEEP level based on the best driving pressure value	Recruiting
Does Automated Closed-Loop Ventilation Reduce the Driving Pressure Levels in Patients With ARDS (AiRDRoP) [NCT03211494]	A randomized crossover clinical trial	Marcus J Schultz (Netherlands)	Automated closed-loop ventilation	Conventional lung protective ventilation	Transpulmonary driving pressure up to day 7	Recruiting
Driving Pressure Limited Ventilation for Patients With ARDS (ART2pilot) [NCT02365038]	A multicenter randomized controlled pilot trial	Alexandre B Cavalcanti (Brazil)	A driving pressure limited mechanical ventilation strategy (driving pressure 13 cmH2O with adjusted tidal volume between 3 and 8 mL/kg of predicted body weight)	The ARDS Clinical Network strategy (tidal volume between 4 and 6 mL/kg of predicted body weight with limited plateau pressure up to 30 cmH2O)	Driving pressure between days 1 and 3	Recruiting

## Conclusion

Mechanical ventilation remains the cornerstone of supportive care for patients with ARDS. While we wait for RCTs confirming the benefit and the prognostic role of the driving pressure in ARDS, lung protective ventilation with lower tidal volumes and inspiratory pressures should be prioritized. Driving pressure can be considered as a complementary tool to adjust tidal volume or PEEP, particularly in patients with severe ARDS.

## Abbreviations

ARDS: Acute respiratory distress syndrome
ICU: Intensive care unit
PEEP: Positive end-expiratory pressure
RCTs: Randomized controlled trials
VILI: Ventilator-induced lung injury
